# Cognitive Control in Young and Older Adults: Does Mood Matter?

**DOI:** 10.3390/brainsci12010050

**Published:** 2021-12-30

**Authors:** Linda Truong, Kesaan Kandasamy, Lixia Yang

**Affiliations:** Department of Psychology, Ryerson University, Toronto, ON M5B 2K3, Canada; ltruong@research.baycrest.org (L.T.); kesaan.kandasamy@ryerson.ca (K.K.)

**Keywords:** proactive control, reactive control, mood, aging, AX-CPT

## Abstract

The dual mechanisms of control framework (DMC) proposes two modes of cognitive control: proactive and reactive control. In anticipation of an interference event, young adults primarily use a more proactive control mode, whereas older adults tend to use a more reactive one during the event, due to age-related deficits in working memory. The current study aimed to examine the effects of mood induction on cognitive control mode in older (ages 65+) compared to young adults (ages 18–30) with a standard letter-cue (Experiment 1) and a modified face-cue AX-CPT (Experiment 2). Mood induction into negative and/or positive mood versus neutral mood was conducted prior to the cognitive control task. Experiment 1 replicated the typical pattern of proactive control use in young adults and reactive control use in older adults. In Experiment 2, older adults showed comparable proactive control to young adults in their response time (RT). Mood induction showed little effect on cognitive control across the two experiments. These results did not reveal consistent effects of mood (negative or positive) on cognitive control mode in young and older adults, but discovered (or demonstrated) that older adults can engage proactive control when dichotomous face cues (female or male) are used in AX-CPT.

## 1. Introduction

Cognitive control is the ability to coordinate and accomplish task goals, particularly in the presence of interfering or conflicting goals [1]. According to the dual mechanisms of control framework (DMC) [2], there are two cognitive control modes that can be engaged to resolve interference. The proactive control mode is an early selection process that actively maintains task context over time to minimize interference, prior to its occurrence. In contrast, the reactive control mode is a late correction process that serves to resolve interference, at the time of its occurrence, through reactivation of task context. As an analogy, using proactive control, you can maintain the goal of buying a book after work throughout the day. However, relying on reactive control, you remember that you need to buy a book only upon seeing the bookstore on your way home.

Proactive and reactive control have been examined using the AX-version of the Continuous Performance Test (AX-CPT) [2,3], in which participants view sequentially presented letter stimuli that serve as valid cues, invalid cues, valid probes, or invalid probes. Each cue is followed by a probe. For 70% of trials, a “target” response is expected in response to a valid probe (“X”), but only when it follows a valid cue (“A”; i.e., AX target trial). Thus, the valid cue becomes a reliable signal for the upcoming valid probe, creating a strong tendency to respond “target” to the forthcoming probe. The remaining 30% of trials are evenly distributed across three non-target trial types: cue-lure (AY), probe-lure (BX), and control (BY). Using proactive control can result in poor performance on cue-lure AY trials (i.e., more errors or longer RT) because the valid cue “A” will lure individuals to prepare a target response, despite being followed by an invalid probe. In contrast, using reactive control can result in poor performance on probe-lure BX trials because individuals are likely to be lured by a valid probe “X” to make a target response, despite the preceding invalid cue “B”. Finally, control trials (BY) do not generate interference as both cues and probes are invalid.

Relative to proactive control, reactive control is less resource-demanding because it is exerted only when needed and does not require continuous maintenance of task context. Past research shows an age-related shift from proactive to reactive control [2,4]. Specifically, older adults perform more poorly on probe-lure than cue-lure trials (signaling reactive control), whereas young adults perform more poorly on cue-lure than probe-lure trials (signaling proactive control).

Past research has examined the effects of mood, reward, and incentive manipulation on cognitive control performance, primarily in young adults [5,6,7,8,9,10]. With regards to the effect of reward, it was revealed that performance-contingent reward (i.e., reward based on task performance) increased the use of cue information and thus facilitated proactive control, whereas random rewards (i.e., unconditional reward not related to performance) reduced proactive control [7,8]. Similarly, penalty-based monetary incentive has been found to lead to increased reactive control [11]. With regard to the mood effect, most previous work focused on the effect of positive mood and revealed mixed results. Some studies found reduced proactive control in positive mood induction [7,8,12]. Similarly, positive affect reduced perseveration (suggesting reduced proactive control) but increased cognitive flexibility and distraction [6]. On the other hand, it was also found that there was a slight increase in proactive control under positive mood relative to neutral mood induction [5]. Chiew and Braver [5] have also found that the reward effect was stronger and more robust than the positive emotion effect, though both tended to enhance proactive control. When affect manipulation occurs on a trial-by-trial basis, positive but not negative mood reduced cue maintenance in AX-CPT [13]. This might be because the positive mood effects were mediated by dopamine that serves to gate information accessibility in cognitive control [14]. It has been demonstrated that dopamine is involved in anticipated punishment avoidance [15]. However, it should be noted that our understanding of the role of dopamine in behavior is still limited and might be contradictory [16]. Nevertheless, the effect of negative mood on cognitive control performance in the AX-CPT remains understudied, even though negative mood could improve task switching performance [17].

Considering the evidence for maintained or even enhanced emotional processing/regulation in older adults [18] and a growing interest in emotion-cognition interactions with aging [19], it is reasonable to predict that mood might impact older adults’ ability to engage in proactive control. However, evidence of the effect of mood/motivation on cognitive control mode in older adults is still rather limited. Given the significant importance of mood in our understanding of human behavior [20], and considering the complexity and inconsistency with regard to the mood effect on cognitive control, the current study aims to specifically examine the effect of negative and/or positive mood induction on the relative engagement of proactive and reactive control in young and older adults.

Additionally, it has been evidenced that older adults’ proactive control could be improved through external environmental (e.g., extended task practice [11]) or cue-oriented strategy training, such as explicit cue-focus instruction in the AX-CPT [4]. Given that face cues have been shown to facilitate recall for older adults [21] and face stimuli are naturally associated with socially meaningful affect, induced mood may facilitate face cue maintenance and thus enhance proactive control use. Thus, the use of face cues in the AX-CPT may provide an optimal condition to detect the mood effect on cognitive control. This face-cue paradigm would also address a prevailing concern of the poor social ecological validity of cognitive control tasks, such as the standard AX-CPT [22], which typically uses only letters. In light of these findings, this study assessed the mood effect on cognitive control in young and older adults in a standard letter-cue (Experiment 1) and a modified face-cue AX-CPT (Experiment 2).

In summary, the current study aimed to systematically examine the effects of mood induction on cognitive control mode in a standard letter-cue and a face-cue AX-CPT in both young and older adults. Specifically, it addresses two related questions: (1) Does negative mood induction modulate cognitive control use in young and older adults in a standard letter-cue AX-CPT (Experiment 1)? (2) Do socially meaningful face cues enhance proactive control and its associated mood effect, particularly in older adults (Experiment 2)? In light of the beneficial effect of reward/penalty on proactive control in older adults [23] and an age-associated shift from a negativity bias to a positivity effect [18] in older adults, we predict that young adults would benefit from negative mood, whereas older adults would benefit from positive mood in proactive control use. Given that contextual or environmental support benefits older adults’ cognitive performance [24,25], we predict the mood benefits in older adults’ proactive control use would be further enhanced by using socially meaningful face cues.

## 2. Experiment 1

Experiment 1 examined the effect of negative mood induction on cognitive control mode with a standard letter-cue AX-CPT in young and older adults. The procedure was approved by the Ryerson Research Ethics Board (REB # 2013-286).

### 2.1. Method

**Sample**. The final sample consisted of 109 participants, including 54 young adults (aged 18–28) and 55 older adults (ages 65–84), who were randomly assigned to a neutral or a negative mood induction condition (see Table 1 for the mean and standard deviation of age for each group). A sensitivity test using G * Power 3.1.9.7 [26] suggested that this sample size allowed a power of 0.85 to detect a moderate main effect (f = 0.25) of a 2-level mood in a between-subjects manipulation at an alpha level of 0.05.

Eligibility criteria included: (a) no history of neurological issues (e.g., stroke, dementia, major head injury); (b) no history of uncontrolled medical conditions (e.g., diabetes, cardiovascular diseases); (c) no current diagnoses of mood disorders (e.g., depression or anxiety disorders) or self-reported “extremely severe” depression/anxiety symptoms with the Depression, Anxiety, and Stress Scale (DASS-21) [27]. Twelve young adults were replaced, including 11 due to high scores on the DASS-21 and 1 due to previous experience of prolonged unconsciousness. Eight older adults were replaced; 2 for high scores (> 6) on the Short Blessed Test (SBT) [28], suggesting possible dementia-related cognitive impairment; 1 for technical issues; 2 for misunderstanding the AX-CPT instructions; and 3 for withdrawal from the experiment. Young adults were recruited from an undergraduate participant pool and were compensated with course credits. Older adults were recruited from the Ryerson Senior Participant Pool (RSPP) and received $20 as an honorarium for their participation. Table 1 displays the sample’s demographic characteristics and cognitive performance.

The 2 (Age: young vs. older) × 2 (Mood: neutral vs. negative) Univariate Analysis of Variance (ANOVA) on each of these demographic and cognitive variables revealed significant age effects in all variables except for health rating (*p* = 0.155, see Table 1). Consistent with literature [29], older adults were higher in positive affect assessed with the Positive and Negative Affect Schedule (PANAS) [30] and vocabulary, but lower in time perspective as assessed with the Future Time Perspective Scale (FTP) [31], PANAS negative affect, depression, stress, and anxiety (DASS-21), as well as all other cognitive measures (*ps* ≤ 0.002). Nevertheless, the two mood groups were largely matched in all variables (*ps* ≥ 0.076), except for education and Pattern Comparison (*ps* ≤ 0.021). Overall, the negative mood group had slightly more years of education and outperformed the neutral mood group on Pattern Comparison (a speed measure). All interactions were not significant (*ps* ≥ 0.065).

**Figure 1:**
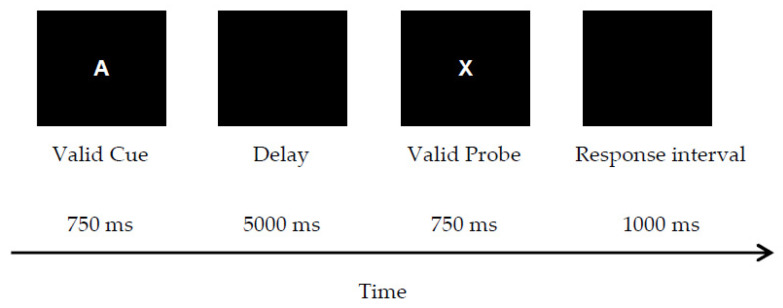
Sample trial procedure for a target trial in the letter-cue AX-CPT (Experiment 1).

**Letter-cue AX-CPT**. The letter-cue AX-CPT modeled previous work [3]. The rule was to press a “target” key to respond to a valid probe (letter X) only when it followed a valid cue (letter A). Invalid cues and probes were all other letters of the alphabet except for K, V, and Y due to their visual similarity to X. All letter stimuli were uppercase, in 36-point bolded white Helvetica font, presented centrally against a black background on a computer monitor. The letter stimuli were sequentially presented one at a time, including the four types of cue-probe pairs. Each cue was presented (750 ms), followed by an unfilled delay (5000 ms). Then, a probe was presented (750 ms), followed by a 1000-ms inter-trial interval (ITI). The response time window was 1750 ms (i.e., probe + ITI). The trial procedure for a target trial is illustrated in Figure 1.

Participants began with a practice block of five trials, followed by two experimental blocks, each including 100 trials: 70 target and 30 non-target trials (10 for each of the non-target trials: cue-lure, probe-lure, and control). The trials were presented in a pseudo-randomized order. Each block lasted for approximately 14 min. Feedback (i.e., correct or incorrect) was provided only for the practice trials.

**Mood inductions and ratings**. The mood induction procedure was adopted from previous work [32], including a slideshow of 30 neutral or low arousal negative pictures selected from the International Affective Picture System (IAPS) [33], each presented centrally on the screen for 5000 ms. The pictures were accompanied by mood-congruent sound clips: ambient street noise for neutral and melancholic classical music for the negative induction. The slideshow lasted 2.5 min and looped once (in a different presentation order) for a total of 5 min for induction. Participants underwent the induction twice, immediately before the first and the second AX-CPT block and rated their current mood state (valence and arousal) at six different time points throughout the task (see the “Overall procedure” section below as well) using the Self-Assessment Manikin (SAM) [34] based on a scale of 1–9, with 1 referring to most negative (i.e., sad) or lowest arousal and 9 denoting most positive (i.e., happy) or highest arousal.

**Overall procedure**. The experiment began with the AX-CPT instructions and the practice block. Then, the first mood rating was collected (pre-induction 1). This was immediately followed by the 5-min neutral or negative mood induction, and then by the second mood rating (post-induction 1). Then, participants completed the first AX-CPT block, followed by the third mood rating (post-block 1). The same procedure was repeated for the second half of the session, including the AX-CPT instruction and a practice block, followed by the second mood induction, and then the second AX-CPT block, with three mood ratings (pre-induction 2, post-induction 2, and post-block 2) integrated accordingly in the same way as in the first block.

The session concluded with a set of affective and cognitive measures to characterize group differences and their potential influences on the AX-CPT performance (see Table 1). Affective measures included the FTP, the PANAS, and the DASS-21. Cognitive tests consisted of a Visual-Spatial Working Memory (VSWM) [35] and four measures from the National Institutes of Health (NIH) Toolbox [36] to measure processing speed (Pattern Comparison), vocabulary (Picture Vocabulary Test), attention/inhibitory control (Flanker Inhibitory Control and Attention Test), and task-switching (Dimensional Change Card Sort Test, DCCST). Older adults were also assessed for potential cognitive impairment using SBT. Finally, participants completed a demographic and health questionnaire. Upon completion, all participants viewed a brief comedic video clip from Just for Laughs Gags as a mood reinstatement. The experiment lasted about 1.5 to 2 h.

**Data analysis**. The statistical significance level was set at α = 0.05. Greenhouse–Geisser corrections were used if Mauchly’s test of sphericity was significant (*p* < 0.05). For follow-up multiple comparisons, a Bonferroni correction was applied.

### 2.2. Results

**Figure 2:**
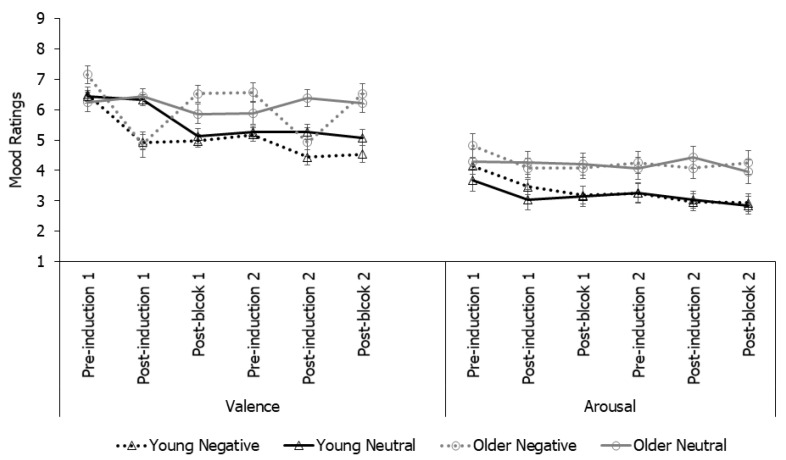
Mean valence and arousal ratings of mood in Experiment 1. Ratings: 1 = most negative (i.e., sad) or lowest arousal; 9 = most positive (i.e., happy) or highest arousal.

**Mood ratings**. As a manipulation check for the effectiveness of a mood induction, SAM valence and arousal ratings were submitted to a 2 (Age) × 2 (Mood) × 6 (Time points: pre-induction 1, post-induction 1, post-block 1, pre-induction 2, post-induction 2, post-block 2) mixed-model ANOVA (Figure 2). The analysis on valence ratings revealed that all main effects and interactions were significant (*ps* ≤ 0.024, ηp^2^ ≥ 0.02) except for mood and the Age by Mood interaction (*ps* ≥ 0.221). Overall, older adults provided higher valence ratings (M = 6.13, SE = 0.16) than young adults (M = 5.33, SE = 0.17), but this age difference was significant only at the final four time points (i.e., from post-block 1 to post-block 2, *ps* ≤ 0.013), primarily because young adults maintained their induced mood state throughout but older adults bounced back to more positive mood following the first block, potentially suggesting more efficient negative mood regulation in older than young adults. Valence ratings were lower in the negative than neutral group at the two critical post-induction time points: at post-induction 1: M = 4.89, SE = 0.21 (negative) and M = 6.37, SE = 0.21 (neutral); at post-induction 2: M = 4.68, SE = 0.23 (negative), and M = 5.83, SE = 0.23 (neutral), *ps* ≤ 0.001, but not significant at all the other time points, *ps* ≥ 0.075. Additionally, only the negative mood group showed a significant drop in valence ratings from pre-induction to post-induction at both induction time points (*ps* ≤ 0.001). These effects were absent in the neutral mood group (*ps* = 1.000). Taken together, the analyses on valence ratings suggested that the mood induction was effective at both time points and for both age groups.

The analysis on arousal ratings revealed an effect of time point (*p* < 0.001, ηp^2^ = 0.06), with arousal level dropping at all subsequent time points relative to baseline (*ps* < 0.055), which may reflect a task familiarity effect. In addition, older adults (M = 4.23, SE = 0.20) rated higher arousal levels than young adults (M = 3.25, SE =0.21), *p* = 0.001, ηp^2^ = 0.10. All other effects were not significant (*ps* ≥ 0.198). The two mood groups did not differ at any time point (*ps* ≥ 0.184).

Taken together, the results suggested successful mood induction. Negative mood induction did reduce the valence ratings for both age groups and at both induction time points. Despite an initial drop, arousal ratings did not vary by mood induction conditions.

**Figure 3:**
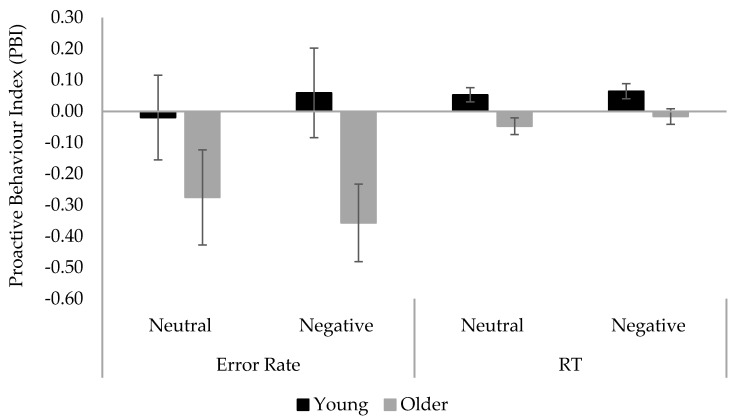
Mean Proactive Behavior Index (PBI) in error rate and RT across age and mood conditions in Experiment 1. Error bars denote standard error.

**Letter-cue AX-CPT performance**. The Proactive Behavior Index (PBI) was calculated using the error rate and reaction times (RT) of correct responses to the critical probe- and cue-lure trials to provide a measure of relative engagement of proactive vs. reactive control [5]. Errors included both incorrect and omitted responses and the error rate was calculated as 1 − accuracy rate. The median RTs were transformed into z-scores for each participant to control for age-related general slowing. The index is calculated as [(cue-lure) − (probe-lure)]/[(cue-lure) + (probe-lure)]. A positive value suggests more engagement in proactive over reactive control, whereas a negative value suggests the opposite. The error rate and RT PBI scores are displayed in Figure 3 and were analyzed with separate 2 (Age) × 2 (Mood) between-subjects ANOVAs. The error rate PBI analysis revealed a significant main effect of age, F(1, 105) = 5.79, *p* = 0.018, MSE = 0.53, ηp^2^ = 0.05. Subsequent One Sample T-tests showed a significant reactive control bias in older adults (M = −0.32, SE = 0.10, *p* = 0.002), but no bias in young adults (M = 0.02, SE = 0.10, *p* = 0.831). All the other effects were not significant (*ps* ≥ 0.566). The RT PBI analysis also revealed significant main effects of age group, F(1, 104) = 13.21, *p* < 0.001, MSE = 0.02, ηp^2^ = 0.11. One Sample T-tests showed a significant proactive control bias in young adults (M = 0.06, SE = 0.02, *p* = 0.001), but a non-significant reactive control bias in older adults (M = −0.03, SE = 0.02, *p* = 0.084). All other effects were not significant (*ps* > 0.395). Taken together, young adults showed a proactive control bias in the RT analysis whereas older adults showed a reactive one in the error rate analysis. The results confirmed the proactive-to-reactive control shift in older adults.

To examine the possible effects of baseline group differences, correlation analyses were performed between relevant demographic or cognitive variables and PBI scores. The results showed that the RT PBI score was positively correlated with FTP score [r(53) = 0.317, *p* = 0.018] and negatively correlated with DASS-21 stress score [r(53) = −0.305, *p* = 0.024] among older adults, whereas the error rate PBI score was positively correlated with DCCST score [r(51) = 0.356, *p* = 0.009] and the RT PBI score was negatively correlated with pattern comparison score [r(50) = −0.331, *p* = 0.017] in young adults. Nevertheless, the main effect of age remained significant in RT PBI score in the corresponding 2 (Age) × 2 (Mood) between-subjects analysis of covariance (ANCOVA), including FTP score (*p* = 0.044), DASS-21 stress score (*p* = 0.001), or pattern comparison score (*p* = 0.006) as covariates. The age effect in error rate PBI, however, disappeared when covarying the DCCST (*p* = 0.916).

To rule out the possible impact of mood induction effectiveness on the lack of the mood effect in the reported results, we reconducted the ANOVA on PBI scores by including only those participants who were relatively successfully induced into the target mood. Specifically in this experiment, we included those with an average post-induction mood rating ≤ 5 in the negative mood induction (18 young and 14 old) and those with an average post-induction mood rating > 5 for the neutral mood condition (19 young and 21 old). The results did not reveal significant effects involving mood (*ps* > 0.374) in both error rate and RT PBI analyses, suggesting that the lack of the mood effect was not likely driven by an unsuccessful mood induction in some participants.

For verification purpose, we also conducted 2 (Age) × 2 (Mood) × 2 (Trial Type: cue-lure vs. probe-lure) mixed model ANOVAs on the original error rate and median RT z-scores (see Table 2). The error rate analysis revealed a significant main effect of trial type, *F*(1, 105) = 14.34, *p* = 0.000, *MSE* = 0.02, ηp^2^ = 0.12, with a higher error rate in probe-lure (M = 0.15, SE = 0.02) than cue-lure trials (M = 0.07, SE = 0.01), suggesting an overarching reactive control bias. All other effects were not significant (F ≤ 1.74, *ps* ≥ 0.190). The RT analysis revealed a significant main effect of age, *F*(1, 104) = 4.15, *p* = 0.044, *MSE* = 0.16, ηp^2^ = 0.04, with a higher error rate in older (M = 0.52, SE = 0.05) than young adults (M = 0.37, SE = 0.05). This was qualified by an Age by Trial Type interaction, *F*(1104) = 14.18, *p* = 0.000, *MSE* = 0.53, ηp^2^ = 0.12. Follow-up multiple comparisons showed that younger adults made more errors to cue-lure (M = 0.60, SE = 0.06) than probe-lure trials (M = 0.14, SE = 0.11), *p* = 0.002, whereas older adults made more errors to probe-lure (M = 0.67, SE = 0.11) relative to cue-lure trials (M = 0.38, SE = 0.06), *p* = 0.042. The results confirmed the PBI analyses for a proactive-to-reactive control shift in older adults.

### 2.3. Discussion

Overall, the mood induction procedure proved to be effective, as the valence ratings dropped only after negative but not neutral mood inductions, at both induction time points and for both age groups. The results were consistent with an earlier study [32] and further validated this mood induction procedure for young and older adults. However, it should be noted that the average post-induction valence ratings fell within the mid-range of the scale. Interestingly, young adults stayed in their post-induction mood throughout the time course whereas older adults’ mood quickly bounced back to more positive ones. Finally, arousal ratings dropped at the initial mood induction, but did not vary by mood induction manipulation. This suggests that the mood induction only effectively changed the mood to the expected valence direction but with little effect on emotional arousal level. The higher baseline arousal level might reflect the task- or anticipation-related anxiety and stress which gradually declined with the progression of the task procedure.

The current experiment showed no evidence for the effect of induced negative mood on cognitive control use. The results are generally consistent with some previous work which did not reveal any effect of negative emotional valence of testing stimuli on cognitive control in young adults [13]. Given the documented effects (though mixed) of positive mood on cognitive control [5,7,8,12], positive mood was predicted to show an effect [13], particularly for older adults given their positivity effect [18]. To test these hypotheses, we added a positive mood induction in Experiment 2 to specifically examine the effects of both positive and negative mood inductions on cognitive control in a modified AX-CPT with socially meaningful face cues.

## 3. Experiment 2

In Experiment 2, we modified the task conditions by using socially meaningful face cues and including positive mood induction to maximize the chance of detecting mood effects, if any, on cognitive control in young and older adults.

### 3.1. Method

**Sample**. A total of 157 participants (80 young and 77 older adults) were randomly assigned to the neutral, negative, and positive mood induction conditions (see Table 3 for the mean and standard deviation of age for each group). A sensitivity test using G * Power 3.1.9.7 suggested that this sample size allowed a power of 0.90 to detect a moderate main effect (f = 0.25) of a 3-level mood between-subjects manipulation at an alpha level of 0.05. The exclusion criteria were the same as in Experiment 1. Table 3 displays the sample characteristics. The 2 (Age) × 3 (Mood) Univariate ANOVA on each of these variables revealed a significant age effect in all variables (*ps* < 0.045). Similar to Experiment 1, older adults were higher in positive affect, health rating, and vocabulary, but lower in FTP, negative affect, depression, stress, and anxiety, as well as all the other cognitive measures. There was a mood by age interaction (*p* = 0.048) in education, with higher education in neutral than in the negative mood group for older (*p* = 0.029), but not for young adults (*p* = 1.00). Additionally, the negative affect score was lowest in the negative mood group, in comparison to other mood groups (*p* ≤ 0.045). All the other variables did not differ across the three mood conditions (*ps* ≥ 0.060). Table 3 displays the sample’s demographic and cognitive profiles.

**Face-cue AX-CPT**.

**Stimuli**. The letter-cue AX-CPT in Experiment 1 was modified by replacing letter cues with color photographs of naturalistic Caucasian adult faces (ages 19–80), with a resolution of 335 × 419 pixels, taken against gray backgrounds, obtained from FACES [37], a validated database. A subset of 32 face images was selected, including 8 young (ages: 19–31) and 8 older (ages 69–80) male or female faces with neutral expressions (two images per person). To ensure the mood effect was driven by the external mood induction manipulation, only faces with neutral expression were included to control for the effect of emotional expressions and the emotional congruency effect. The faces across age by gender categories were counterbalanced and equally assigned to serve as valid and invalid cues and they were centrally displayed against a black background on the computer screen.

**Figure 4:**
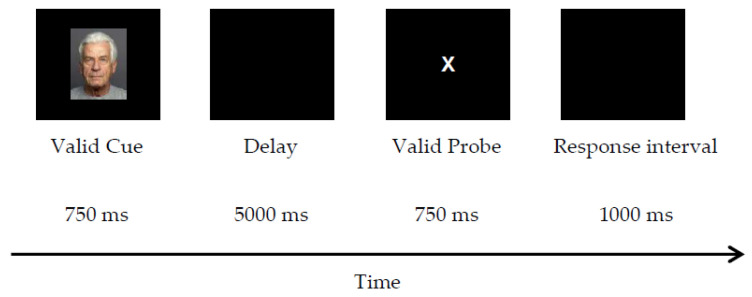
Sample trial procedure for a target trial in the face-cue AX-CPT (Experiment 2).

**Trial procedure**. The trial procedure and structure modeled Experiment 1 (Figure 4), except the cue stimuli were faces instead of letters. What constituted a valid cue was based on gender classification (either male or female faces as valid cues). Specifically, the valid cues were male faces in one block (valid-male) and female faces in the other block (valid-female). This was to control for potential gender preference or practice effects related to the specific mapping between a gender and the cue identity. The order of the two blocks was counterbalanced across participants. A target response was required to the letter X (valid-probe) only if it followed a valid face cue (female or male in each block). Cue-lure trials consisted of a valid face cue followed by an invalid letter probe. Probe-lure trials consisted of an invalid face cue followed by a valid-letter probe “X”. Control trials consisted of an invalid face cue followed by an invalid letter probe.

**Overall procedure**. The procedure was identical to that of Experiment 1 except for the addition of a positive mood induction condition in which participants viewed positive images against calm/peaceful classical music background during the mood induction.

### 3.2. Results

**Figure 5:**
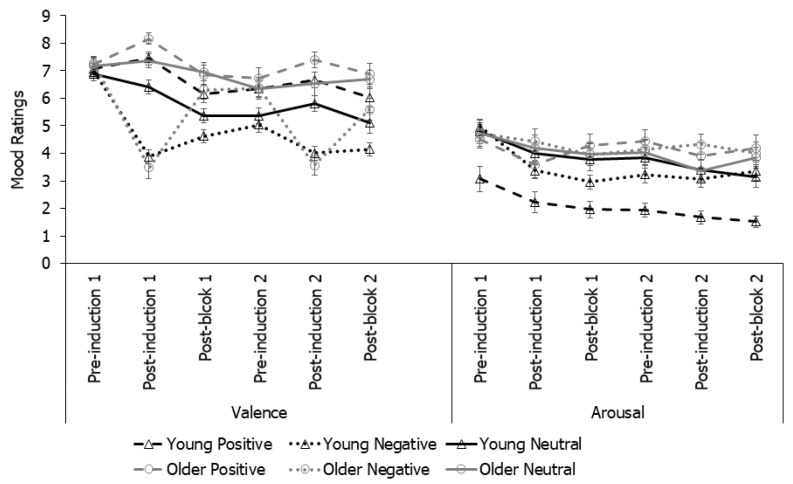
Mean valence and arousal ratings of mood in Experiment 2. Ratings: 1 = most negative (i.e., sad) or lowest arousal; 9 = most positive (i.e., happy) or highest arousal.

**Mood ratings**. The 2 (Age) × 3 (Mood) × 6 (Time point) mixed ANOVA on valence ratings (Figure 5) revealed that all main effects and interactions were significant (*ps* ≤ 0.004, ηp^2^ ≥ 0.03) except for the Age by Mood interaction (*p* = 0.632). Overall, older adults provided higher valence ratings (M = 6.49, SE = 0.14) than young adults (M = 5.74, SE = 0.14), *p* < 0.001, ηp^2^ = 0.09). As predicted, higher valence ratings were made by those in the positive (M = 6.91, SE = 0.17), than neutral (M = 6.33, SE = 0.17), and then followed by negative mood (M = 5.10, SE = 0.17) group (*ps* ≤ 0.050). It should be noted that age differences were significant only at the later time points (i.e., two post-block time points and pre-induction 2, *ps* ≤ 0.001). Specifically, in negative and neutral conditions, young adults held the induced mood whereas older adults quickly bounced back to baseline, suggesting more efficient negative mood regulation in older than young adults. It revealed a higher valence rating in positive than neutral group only at both post-induction time points (*ps* ≤ 0.021). In contrast, there was a lower rating in the negative than neutral group only at the two post-induction time points (*ps* ≤ 0.001) and after the second AX-CPT block (*p* = 0.013). On the other hand, the negative mood group showed a significant drop at both post-induction time points (*ps* ≤ 0.001), whereas the positive mood group tended to increase valence ratings at both post-induction time points, but the effect was approaching significance at the first (*p* = 0.053) but not the second post-induction time point (*p* = 0.155). The neutral mood group did not change at both post-induction time points (*ps* = 1.000). Taken together, these analyses suggested that the mood inductions were effective at inducing the intended moods for both age groups.

The same analysis on arousal ratings revealed a time point effect (*p* < 0.001, ηp^2^ = 0.10), qualified by an Age by Time Point interaction (*p* = 0.036, ηp^2^ = 0.02). There were also main effects of age group (*p* < 0.001, ηp^2^ = 0.11) and mood group (*p* = 0.009, ηp^2^ = 0.07), qualified by an Age by Mood interaction (*p* = 0.004, ηp^2^ = 0.07). The arousal ratings dropped at almost all subsequent time points relative to baseline (*ps* < 0.001). Older adults (M = 4.15, SE = 0.17) rated a higher arousal level than young adults (M = 3.13, SE = 0.17), *p* = 0.001, and this age effect was seen across all time points (*ps* ≤ 0.009) except at baseline (*p* = 0.226). For young, but not older adults, the positive mood group rated a lower arousal level than negative or neutral mood groups (*ps* ≤ 0.002), but the latter two did not differ (*p* = 1.00). The lower arousal level in young adults with positive mood induction may reflect a random group difference present even before the mood induction was introduced (i.e., pre-induction 1).

Similar to Experiment 1, these results suggested that the mood inductions were largely effective. Negative mood induction reduced whereas positive mood induction increased valence ratings for both age groups, despite a universal arousal drop after induction.

**Figure 6:**
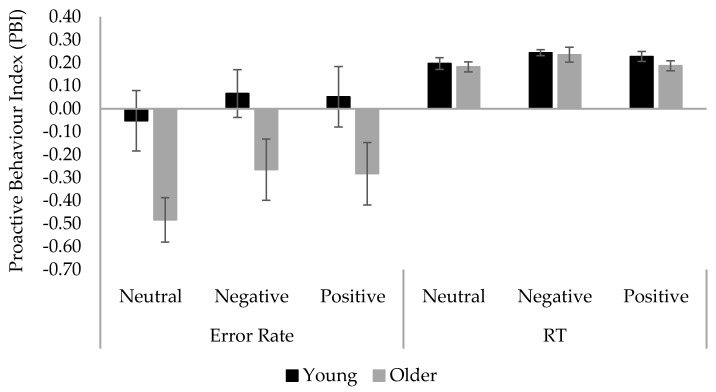
Mean Proactive Behavior Index (PBI) in error rate and RT across age and mood conditions in Experiment 2. Error bars denote standard error.

**Face-cue AX-CPT performance**. The error rate and RT PBI scores were calculated and analyzed in the same way as in Experiment 1 (see Figure 6). The error rate PBI analysis revealed a significant main effect of age, F(1, 151) = 13.11, *p* < 0.001, MSE = 0.40, ηp^2^ = 0.08. Subsequent One Sample T-tests showed a significant reactive control bias in older adults (M = −0.34, SE = 0.07, *p* < 0.001), but no bias in young adults (M = 0.02, SE = 0.07, *p* = 0.758). All the other effects were not significant (*ps* ≥ 0.326). The RT PBI analysis did not reveal any significant effects (*ps* ≥ 0.100). Both age groups showed a significant proactive control bias (young: M = 0.22, SE = 0.01; older: M = 0.20, SE = 0.02; *ps* ≤ 0.001). These results are displayed in Figure 6. Correlation analyses showed that error rate PBI was negatively correlated with DASS-21 anxiety score, r(74) = −0.228, *p* = 0.048, in older adults, but positively correlated with DCCST score, r(78) = 0.239, *p* = 0.035, in young adults. The main effect of age remained unchanged in the subsequent two-way ANCOVAs on error rate PBI covarying DASS-21 anxiety (*p* = 0.010) or DCCST score (*p* = 0.048).

To rule out the possible impact of mood induction effectiveness, we included those with an averaged post-induction mood rating ≤ 5 for the negative mood condition (24 young and 23 old), a rating ≥ 7 for the positive mood condition (19 young and 20 old), and a rating between 5 and 7 for the neutral mood condition (14 young and 5 old). Similar to Experiment 1, the results did not reveal any significant effects involving mood (*ps* > 0.230) in both error rate and RT analyses. These results showed that the lack of the mood effect was not likely driven by an unsuccessful mood induction in some participants.

To examine whether the face-cue task (Experiment 2) elicited more proactive control engagement in older adults relative to the letter-cue task (Experiment 1) in the RT analysis, a cross-experiment 2 (Age) × 2 (task: letter-cue vs. face-cue) ANOVA was run on the RT PDI scores. The results showed significant main effects of age, F(1, 206) = 8.53, *p* = 0.004, MSE = 0.02, ηp^2^ = 0.04, and task, F(1, 206) = 134.43, *p* < 0.001, MSE = 0.02, ηp^2^ = 0.40, qualified by an Age by Task interaction, F(1, 206) = 5.33, *p* = 0.022, MSE = 0.02, ηp^2^ = 0.03. Young adults showed a higher proactive control index than older adults in the letter-cue (*p* < 0.001), but not in the face-cue task (*p* = 0.670), suggesting that the use of the face cues did benefit older adults, through engagement of proactive control to the same degree as young adults.

For verification purposes, we also conducted 2 (Age) × 3 (Mood) × 2 (Trial Type: cue-lure vs. probe lure) mixed model ANOVAs on the error rate and median RT z-scores (see Table 4). The error rate analysis revealed a significant main effect of trial type, F(1, 151) = 15.93, *p* = 0.000, MSE = 0.03, ηp^2^ = 0.10, with a higher error rate in probe-lure (M = 0.16, SE = 0.02) than cue-lure trials (M = 0.09, SE = 0.01), suggesting an overall reactive control bias. All other effects were not significant (*ps* ≥ 0.109). The RT analysis revealed a significant main effect of trial type, F(1147) = 540.70, *p* = 0.000, MSE = 0.28, ηp^2^ = 0.79, with longer RTs in cue-lure (M = 0.76, SE = 0.04) than probe-lure trials (M = −0.64, SE = 0.04). The main effect of age was also significant, F(1147) = 4.19, *p* = 0.043, MSE = 0.09, ηp^2^ = 0.03, with slower RTs in older (M= 0.11, SE = 0.04) than young adults (M = 0.01, SE = 0.03). There was also a Trial Type by Mood interaction, F(1147) = 3.43, *p* = 0.035, MSE = 0.28, ηp^2^ = 0.05. Although the trial type effect was significant for all mood conditions, the difference between cue-lure and probe-lure trials was larger in negative (1.61) than positive (1.34) or neutral (1.24) mood conditions. All other effects were not significant (*ps* ≥ 0.056). Despite the reactive control tendency in the error rate analysis, the RT results largely confirmed the PBI analysis for a proactive control bias in both age groups.

### 3.3. Discussion

Similar to Experiment 1, despite a universal drop in arousal, the mood inductions were validated as effective at inducing the expected valence. Replicating and expanding Experiment 1, older adults were faster than young adults in regulating out of the induced negative mood following the induction.

Older adults demonstrated a consistent reactive control bias across error rate and RT PBI analysis in the letter-cue AX-CPT task of Experiment 1. However, this pattern was replicated only in the error rate PBI analysis but absent in the RT PBI analysis in the face-cue AX-CPT of Experiment 2. Specifically, the RT PBI analysis showed an equivalent proactive over reactive control bias for both young and older adults, suggesting that face cues might be sufficiently powerful for older adults to engage proactive control to a level largely comparable to that of young adults. Given that RT measures appear more sensitive at capturing the reactive control tendency in older adults [38], as well as in consideration of the possible floor effects in error rate (M = 0.10–0.13 across the two blocks in each of the two experiments), RT data were prioritized in drawing conclusions whenever there was a discrepancy between error rate and RT analyses.

However, the results showed minimal or no effects of mood (positive or negative) on cognitive control, despite previous evidence for effects of positive mood/valence or motivation/reward on cognitive control [23]. Replicating Experiment 1, negative mood did not affect cognitive control either.

## 4. Discussion

This study aimed to examine the effects of mood on cognitive control in young and older adults. The results of Experiment 1 replicated the proactive and reactive bias in young and older adults, respectively, with a standard letter-cue AX-CPT. Using a more socially meaningful face-cue AX-CPT, Experiment 2 revealed an age-equivalent proactive control bias in the RT analysis. Nevertheless, cognitive control did not seem to vary by mood induction. It should be noted, however, that similar to previous work [1,4,38,39], the age effect varied across RT and error rate analyses.

### 4.1. Mood Manipulation

The mood rating analyses aimed to validate the effectiveness of the mood induction manipulation in differentially changing mood into the expected valence direction for both young and older adults [32]. Across experiments and induction conditions, it was observed that young adults stayed in their post-induction mood throughout the time course whereas older adults’ mood quickly returned closer to baseline. This may suggest that older adults are generally faster in mood regulation [40]. Across both experiments, participants started with a higher baseline arousal which gradually dropped across time. The higher baseline arousal level may reflect the task- or anticipation-related anxiety and stress which gradually declined with practice and the progression of the task procedure, due to habituation and/or increased procedural familiarity with the task.

### 4.2. Age Differences in Cognitive Control

Consistent with the DMC, older adults showed a reactive control bias, compared to young adults who showed a proactive control bias in the standard AX-CPT. This age effect remained intact after controlling for most related demographic and background variables (e.g., FTP or DASS scores). It should be noted that the age effect disappeared when covarying DCCST score (in Experiment 1), possibly due to the overlaps in conceptual structure and performance requirements between DCCST and AX-CPT [41]. Similar to some previous works [2,4,38,39], age differences varied across RT and error rate data. For example, Braver and colleagues [38] showed that young adults committed greater cue-lure errors relative to older adults (indicative of proactive control), but reactive control in older adults is typically observed in RT data, through a relatively delayed response to probe-lure (BX) trials as task goals are largely reactivated upon probe presentation.

However, the results of Experiment 2 indicated that this age-related decline in proactive control is partially amenable. Though the exact mechanisms are unclear, we offer two speculations: (a) the marked distinction between a face cue and a letter probe may have directed processing towards the cue and away from the probe; (b) the dichotomous nature (female or male) of face cues may have made it easier to process and maintain the cue/contextual information. In support of these speculations, past studies have shown that cognitive control mode changes with task demands. For example, individuals with high working memory capacity can behave in a reactive manner when a high proportion of cues become invalid [42]. Finally, face cues may elicit deeper processing in the brain relative to letter cues, considering greater activation observed when preparing to remember facial cues in a face working memory paradigm [43]. It has been shown that proactive control is associated with increased and sustained activity in the anterior prefrontal cortex [44]. Taken together, the results suggested that face cues were probably sufficiently powerful in directing subsequent responses and thus helped older adults sustain proactive control processes. However, these speculations need to be further tested.

### 4.3. Mood and Cognitive Control

Although research has revealed significant (though mixed) effects on cognitive control of positive mood/valence and rewards in young adults [5,9] and the motivation/reward effects in older adults [23], the effect of mood (negative or positive) is minimal or absent in the current study. The lack of a negative mood effect is inconsistent with the result of improved task switching performance under negative mood [17]. However, it is somewhat consistent with earlier literature [13]. This has been explained by the lack of relationship between negative affect and dopamine, which is assumed to underlie information regulation in cognitive control [14], though our understanding of the exact role of dopamine in behavior is still limited [15,16].

Despite an established relationship between positive mood and dopamine [45] as well as the age-associated positivity effect [18], the current study did not find any significant effect of positive mood on cognitive control in either young or older adults. This seems to be inconsistent with other studies that have found emotion effects in older adults on processes that are presumably related to cognitive control [32,46,47] and those that have found reduced proactive control in young adults under positive mood or performance-contingent reward [7,8,12,13]. For this, we provide the following speculations: (1) Although both motivation/reward and mood manipulations were proposed to modulate cognitive control, it is possible that motivation might be more sensitive relative to mood induction, as there is evidence that reward does modulate proactive control in older adults [23]. This speculation is also supported by previous work which found a reward effect was more robust than a mood effect among young adults [5]; (2) It is possible that the behavior measures used in this study were not sufficiently sensitive to detect any subtle mood effects; and (3) it is possible that the socially meaningful face cues in Experiment 2 overshadowed any mood effects. These speculations, however, need to be further tested in future studies.

### 4.4. Limitations

A couple of limitations should be noted. The first is about the mood induction procedure and measures. It is possible that the induction procedure may have only induced low-arousal mood which might be too mild to exert any effect on cognitive control. In future studies, use of continuous psychophysiological measures of mood (e.g., heart rate or electromyography) would be advantageous. Second, mood was manipulated in a between-subjects design; it is possible that pre-existing group differences in cognitive control may have masked performance differences following the induction. We did not use within-subjects manipulation of mood induction, to minimize possible effects due to fatigue [5] and confounding practice [4]. Third, the effect of using face cues might be confounded with other factors, such as the dichotomous nature of the cues (male vs. female faces), as well as reduced task demand. Fourth, use of faces with a neutral expression might have limited the possibility of detecting mood effects. Future studies may follow up by using faces with emotional expressions in the task.

## 5. Conclusions

Nevertheless, although the mood effect was absent, the current study provided some promising evidence that older adults’ proactive control could be improved through task-specific manipulations, such as using socially meaningful dichotomous face cues. Elucidating factors that can attenuate age-related declines has valuable implications for interventions, particularly as they relate to neurodegenerative disease. Specifically, given the known involvement of reactive and proactive inhibitory control processes in directing cessation or adaptive behaviors, respectively, in Parkinson’s disease [48], it may be interesting to compare cognitive control and inhibitory processes in aging. Future work may help identify similar compensatory mechanisms or environmental modifications that could be applied to facilitate older adults’ cognitive and everyday functioning.

## Figures and Tables

**Table 1:** Experiment 1 participant demographic characteristics and cognitive performance.

Characteristic	Young Adults				Older Adults			
	Neutral Mood (*n* = 26)		Negative Mood (*n* = 28)		Neutral Mood (*n* = 28)		Negative Mood (*n* = 27)	
	M	SD	M	SD	M	SD	M	SD
Age	19.12	2.03	20.32	3.00	71.21	4.90	70.74	5.22
F/M (ratio)	22/4		26/2		16/12		19/8	
Years of formal Education ^a,b^	12.85	1.99	14.11	1.72	15.63	3.11	17.00	4.24
Health rating	7.90	0.80	7.73	1.14	8.21	1.37	8.13	1.67
FTP ^a^	54.58	6.29	52.32	7.66	39.54	10.86	43.19	9.58
PANAS-PA ^a^	25.15	8.18	22.96	4.91	33.93	6.72	36.48	6.47
PANAS-NA ^a^	16.08	4.20	14.82	4.29	11.89	1.99	12.93	3.69
DASS-Dep ^a^	6.92	5.07	7.43	5.98	3.86	4.77	4.37	4.00
DASS-Anx ^a^	7.69	5.71	7.14	5.97	2.50	3.38	3.04	3.70
DASS-Strs ^a^	13.54	7.64	11.57	6.83	7.29	6.89	6.81	5.64
VSWM ^a^	0.64	0.15	0.64	0.14	0.34	0.18	0.32	0.19
Pattern Comparison * ^a,b^	74.73	10.74	81.00	8.52	49.31	8.84	52.04	11.53
DCCST * ^a^	9.18	0.59	9.29	0.64	7.57	1.18	8.08	0.98
Flanker * ^a^	9.62	0.38	9.54	0.30	8.45	0.66	8.72	0.64
Vocabulary * ^a^	1539.88	111.01	1582.11	155.18	2047.00	576.31	1965.71	220.86
SBT					0.79	1.00	0.22	0.16

Note. M = mean; SD = standard deviation; F/M (ratio) = female/male ratio; Health rating refers to self-rated health status on a scale from 1 (poor) to 10 (excellent); FTP = Future Time Perspective Scale; PANAS-PA/NA = Positive Affect/Negative Affect scores on the Positive and Negative Affect Schedule (PANAS); DASS-Dep/Anx/Strs = Depression, Anxiety, and Stress scores on the DASS-21; VSWM = Visual Spatial Working Memory task; DCCST = Dimensional Change Card Sort Test (task-switching); Flanker = Flanker Inhibitory Control and Attention Test; Vocabulary was measured with Picture Vocabulary Test; SBT = Short Blessed Test. * Measures from the NIH Toolbox (Northwestern University and the National Institutes of Health, 2012). ^a^ Significant main effect of age group (*ps* ≤ 0.002); ^b^ Significant main effect of mood group (*ps* ≤ 0.023) in the 2 (Age) × 2 (Mood) Univariate ANOVAs. The interaction was not significant in all comparisons.

**Table 2:** Mean error rates and RT median z-scores of the critical cue-lure and probe-lure trials, as well as the PBI scores across age and mood conditions in Experiment 1.

Conditions	Young Adults			Older Adults		
	Probe-Lure	Cue-Lure	PBI	Probe-Lure	Cue-Lure	PBI
Error rate						
Neutral	0.14 (0.21)	0.08 (0.08)	−0.02 (0.69)	0.16 (0.22)	0.04 (0.08)	−0.28 (0.80)
Negative	0.15 (0.18)	0.10 (0.08)	0.06 (0.76)	0.13 (0.23)	0.05 (0.11)	−0.36 (0.64)
RT						
Neutral	0.19 (0.65)	0.61 (0.43)	0.05 (0.11)	0.78 (0.97)	0.35 (0.30)	−0.05 (0.14)
Negative	0.09 (0.71)	0.57 (0.50)	0.06 (0.13)	0.56 (0.87)	0.42 (0.43)	−0.02 (0.13)

Note: Mean scores with standard deviations presented in parentheses.

**Table 3:** Experiment 2 Participant Demographic Characteristics and Cognitive Performance.

Characteristic	Young Adults						Older Adults					
	Neutral Mood (*n* = 27)		Negative Mood (*n* = 26)		Positive Mood (*n* = 27)		Neutral Mood (*n* = 25)		Negative Mood (*n* = 27)		Positive Mood (*n* = 25)	
	M	SD	M	SD	M	SD	M	SD	M	SD	M	SD
Age	20.63	3.00	20.62	2.73	21.04	3.20	74.04	6.84	72.00	4.90	73.44	7.33
F/M (ratio)	22/5		20/6		23/4		20/5		24/3		21/4	
Years of formal education ^aXb^	14.07	1.67	14.00	2.18	14.41	1.59	16.94	3.04	14.85	2.33	15.28	3.09
Health rating ^a^	7.82	1.39	7.85	1.05	7.65	1.28	8.25	1.11	8.33	1.41	8.92	0.93
FTP ^a^	51.78	8.47	50.65	7.57	51.89	9.61	38.12	7.39	38.56	11.15	40.48	12.03
PANAS-PA ^a^	24.74	5.47	24.08	8.05	26.00	7.64	35.00	5.59	35.67	6.09	38.92	5.85
PANAS-NA ^a,b^	14.59	3.43	14.96	3.45	12.15	3.34	12.28	2.97	13.63	5.81	12.04	3.30
DASS-Dep ^a^	7.00	6.48	8.38	6.50	7.26	5.44	4.58	4.66	5.19	5.69	4.64	4.99
DASS-Anx ^a^	8.00	5.38	7.08	4.57	6.81	5.58	2.56	3.98	3.56	4.24	2.75	2.88
DASS-Strs ^a^	13.33	7.96	13.31	7.90	12.44	8.31	7.12	5.36	9.11	5.91	9.36	6.50
VSWM ^a^	0.50	0.14	0.61	0.15	0.58	0.29	0.25	0.14	0.27	0.15	0.29	0.21
Pattern Comparison ^a^	71.56	14.38	67.81	47.44	71.19	11.29	45.32	9.53	47.44	9.92	47.74	9.62
DCCST ^a^	8.84	1.19	9.04	0.64	9.06	0.55	7.75	0.84	7.59	1.17	7.73	1.14
Flanker ^a^	9.42	0.52	9.42	0.53	9.50	0.44	8.53	0.53	8.50	0.81	8.34	0.82
Vocabulary ^a^	1475.22	217.17	1497.46	211.78	1551.15	139.67	1925.56	250.78	1930.37	226.87	2082.00	612.66
SBT	20.63						0.40	0.20	0.74	1.13	0.96	1.65

Note: M = mean; SD = standard deviation; F/M (ratio) = female/male ratio; Please refer to the note of Table 1 for the denotations of each variable. ^a^ Significant main effect of age group (*ps* < 0.045); ^b^ Significant main effect of mood group (*ps* < 0.016); ^aXb^ Significant Age by Mood interaction (*p* = 0.048) in the 2 (Age) × 3 (Mood) Univariate ANOVAs.

**Table 4:** Mean error rates and RT median z-scores of the critical cue-lure and probe-lure trials, as well as the PBI scores across age and mood conditions in Experiment 2.

Conditions	Young Adults			Older Adults		
	Probe-Lure	Cue-Lure	PBI	Probe-Lure	Cue-Lure	PBI
Error rate						
Neutral	0.23 (0.27)	0.14 (0.11)	−0.05 (0.68)	0.17 (0.22)	0.05 (0.08)	−0.48 (0.48)
Negative	0.12 (0.19)	0.11 (0.12)	0.07 (0.53)	0.15 (0.22)	0.07 (0.09)	−0.26 (0.69)
Positive	0.15 (0.24)	0.09 (0.09)	0.05 (0.68)	0.13 (0.15)	0.07 (0.08)	−0.26 (0.68)
RT						
Neutral	−0.56 (0.57)	0.65(0.41)	0.20 (0.13)	−0.56 (0.58)	0.71 (0.40)	0.18 (0.11)
Negative	−0.85 (0.27)	0.75 (0.28)	0.24 (0.07)	−0.59 (0.56)	1.00 (0.71)	0.24 (0.16)
Positive	−0.69 (0.43)	0.71 (0.36)	0.23 (0.11)	−0.60 (0.59)	0.67 (0.37)	0.19 (0.11)

Note: Mean scores with standard deviations presented in parentheses.

## Data Availability

The final data and analysis files (in SPSS) could be retrieved from https://doi.org/10.17605/OSF.IO/ZGPR2 (accessed on 11 November 2021).
